# Clinical Spectrum of Infections Among People Who Inject Drugs, With Special Reference to Infective Endocarditis

**DOI:** 10.7759/cureus.99336

**Published:** 2025-12-15

**Authors:** Aktar Hussain, Anjan J Talukdar, Subhajit Mitra, Sangitanjan Dutta, Chiranjita Phukan, Sushmita Singha

**Affiliations:** 1 Medicine, Gauhati Medical College and Hospital, Guwahati, IND; 2 Internal Medicine, Gauhati Medical College and Hospital, Guwahati, IND

**Keywords:** clinical spectrum, infection, infective endocarditis, people who inject drugs, public health

## Abstract

Background

Injection drug use (IDU) is a significant public health concern worldwide and is frequently associated with serious health complications, including infectious diseases. Among these, infective endocarditis (IE) stands out as a particularly concerning infection, posing a significant threat to people who inject drugs (PWID). This study examines the clinical spectrum of infections, with a specific focus on IE among PWID.

Methods

This was a hospital-based cross-sectional study conducted over a period of one year to assess subjects comprising PWID. Study participants were recruited from both the inpatient and OPDs of GMCH. Data were collected using a predesigned proforma, and analysis was performed using IBM SPSS Statistics for Windows, Version 25.0 (Released 2017; IBM Corp., Armonk, NY, USA).

Results

Our findings indicate that young adults are increasingly represented among PWID, with a mean age of 25.94 years and a predominance of males. Analysis of substances used by PWID highlights heroin as the predominant drug. The study reports a high prevalence of psychiatric comorbidities and concomitant hepatitis C infection among PWID. Overall, skin and soft tissue infections (SSTIs; 36%) emerged as the most commonly acquired injection-related infection among PWID, and IE comprised 18% of the presenting infections.

Conclusions

The high frequency of IE among patients with SSTIs and hepatitis C virus highlights the critical need for improved screening using blood cultures and echocardiography in young PWID. Targeted interventions are necessary to enhance the diagnosis and management of IE, which could slow the progression from SSTIs to IE and reduce associated mortality and morbidity. Ultimately, these findings improve our understanding of the clinical spectrum of infections among PWID.

## Introduction

Injection drug use (IDU) remains a significant public health concern, with approximately 15.6 million people aged 15-64 years injecting drugs worldwide [1]. This practice exposes individuals to various health risks, including infectious complications such as HIV, hepatitis B virus (HBV), hepatitis C virus (HCV), and bacterial infections such as infective endocarditis (IE) [2]. The prevalence of IDU varies across regions and populations, including urban areas and marginalized communities [3]. In addition to geographical disparities, IDU is influenced by socioeconomic factors, including poverty, unemployment, and lack of access to health care services [4]. Social determinants of health play a crucial role in shaping patterns of drug use and the associated risks of infectious complications [5].

Injecting drugs poses unique challenges compared with other routes of drug administration, primarily due to the direct introduction of pathogens into the bloodstream through contaminated needles and syringes [6], thereby bypassing the body’s natural defense mechanisms and increasing the risk of blood-borne infections [7]. According to recent estimates, approximately 13.1 million people who inject drugs (PWID) are living with HCV, representing a substantial global disease burden [8]. The sharing of injection equipment, including needles, syringes, and drug preparation paraphernalia, amplifies the risk of transmission of blood-borne pathogens within networks of PWID [9].

IDU is associated with an increased risk of bacterial infections, including IE, a serious infection of the endocardial surface of the heart [10]. IE may occur as a result of bacteremia secondary to skin and soft tissue infections (SSTIs), which are common among PWID [11]. The most common causative agents of IE among PWID include *Staphylococcus aureus *and *Streptococcus *species, with *S. aureus *being predominant in cases associated with IDU [12]. The pathogenesis of IE among PWID is multifactorial, involving the introduction of bacteria into the bloodstream through contaminated injection sites, colonization of the cardiac endothelium, and the formation of microbial vegetations on heart valves [13].

The clinical presentation of IE among PWID can vary widely, ranging from subtle symptoms such as fever and malaise to more severe complications, including septic emboli and valvular dysfunction [14]. Prompt diagnosis and treatment are essential to prevent complications and improve outcomes; however, diagnosing IE among PWID can be challenging due to atypical presentations, overlapping clinical features with other conditions, and limited access to health care services [12,15]. Management of IE often requires a multidisciplinary approach, including antimicrobial therapy tailored to the causative organism, surgical intervention for complications such as valve regurgitation or abscess formation, and long-term follow-up to monitor for relapse or reinfection [16].

Preventing infectious complications among PWID requires a comprehensive approach that addresses both individual- and structural-level factors contributing to IDU and its associated risks [17]. Harm reduction strategies, including needle exchange programs, opioid substitution therapy (OST), and education on safer injection practices, have been shown to reduce the risk of blood-borne infections and improve health outcomes among PWID [18]. However, effective implementation of these interventions requires collaboration among health care providers, policymakers, law enforcement agencies, and community organizations to address the complex social, economic, and environmental factors driving IDU and its associated harms [19].

The purpose of this study is to address gaps in knowledge by examining the clinical spectrum of infections among PWID, with a specific focus on IE. The findings will contribute to the existing literature by providing insights and strategies to improve the diagnosis and management of IE and other infectious complications in this high-risk population.

## Materials and methods

Study center

This was a hospital-based cross-sectional study conducted in Assam, India. A cross-sectional design was used to allow the collection of data from a representative sample of PWID between October 1, 2022, and September 30, 2023.

Inclusion and exclusion criteria of study participants

The inclusion criteria comprised PWID aged 18-65 years. The exclusion criteria included PWID aged <18 years or >65 years, patients with a history of blood transfusion, and patients with predisposing cardiac conditions, including congenital heart disease, rheumatic heart disease, prosthetic heart valves, and degenerative or acquired valvular conditions.

Methodology

After obtaining written informed consent from eligible participants, data were collected using a predesigned proforma encompassing multiple domains, including epidemiology, clinical examination, relevant investigations, and clinical outcomes.

Investigations

A comprehensive array of investigations was conducted to assess the clinical status and infectious profile of participants, including a complete hemogram; biochemistry tests (including liver and kidney function tests); viral markers (HBsAg, anti-HCV, and HIV 1/2); radiological investigations (including chest X-ray); urine routine examination and culture; blood culture; and two-dimensional echocardiography. Patients were classified as having “possible,” “definite,” or “rejected” IE according to the Modified Duke criteria (2000).

Data collection

Data were collected through structured interviews and medical record reviews. Standardized questionnaires were used to collect information on demographic characteristics, drug use history, injection practices, and clinical symptoms. In addition, relevant medical records, laboratory reports, and imaging studies were reviewed to obtain detailed clinical data, including diagnostic test results and treatment outcomes.

Study procedure

The study commenced with the recruitment of eligible participants presenting to the casualty department, OPD, or inpatient wards. Eligibility was determined based on the inclusion and exclusion criteria. Participants identified as PWID, IDU, or IV drug users (IVDU) were considered for study purposes. According to the National AIDS Control Programme Phase III, these individuals are defined as persons with a history of injecting drug use at least once in the past three months. The UNAIDS and WHO definitions of PWID include individuals who inject non-medically sanctioned psychoactive or psychotropic substances. These substances include opioids, amphetamines, cocaine, hypnotics, and sedatives and may be administered via IV, intramuscular, subcutaneous, or intradermal routes.

Data analysis

The collected data were analyzed using appropriate statistical methods. Descriptive statistics, including frequencies, percentages, means, and SDs, were used to summarize demographic characteristics, clinical features, and study outcomes. Inferential statistics, such as chi-square tests and logistic regression analysis, were employed to explore associations between variables and identify predictors of IE outcomes.

Ethical considerations

Ethical approval was obtained from the Institutional Ethics Committee (permission number: MC/190/2007/pt-11/Sept.2022/45) before commencement of the study. Ethical principles outlined in the Declaration of Helsinki and relevant national guidelines were strictly adhered to throughout the study period, ensuring that the welfare and rights of all participants were protected.

## Results

Demographic profile of study participants

The study sample consisted of 50 individuals with a mean age of 25.94 years (Table 1). An SD of 6.498 indicates moderate variability in age, suggesting that most participants were in their mid-20s. The age range spanned 26 years, indicating considerable variation in participant ages, likely ranging from the late teens to early 40s.

**Table 1:** Demographic profile of the study participants

Demographic variable	Statistic/frequency	Value (%)
Age (years)	Mean	25.94
	SD	6.49
	Range	26
Gender	Male	44 (88)
Total	Female	6 (12)
		50 (100)

The gender distribution showed a marked predominance of males among PWID, with 88% (44 of 50 participants) being male and 12% (6 of 50 participants) being female (Table 1).

Substances used among study participants and OST

**Figure 1:**
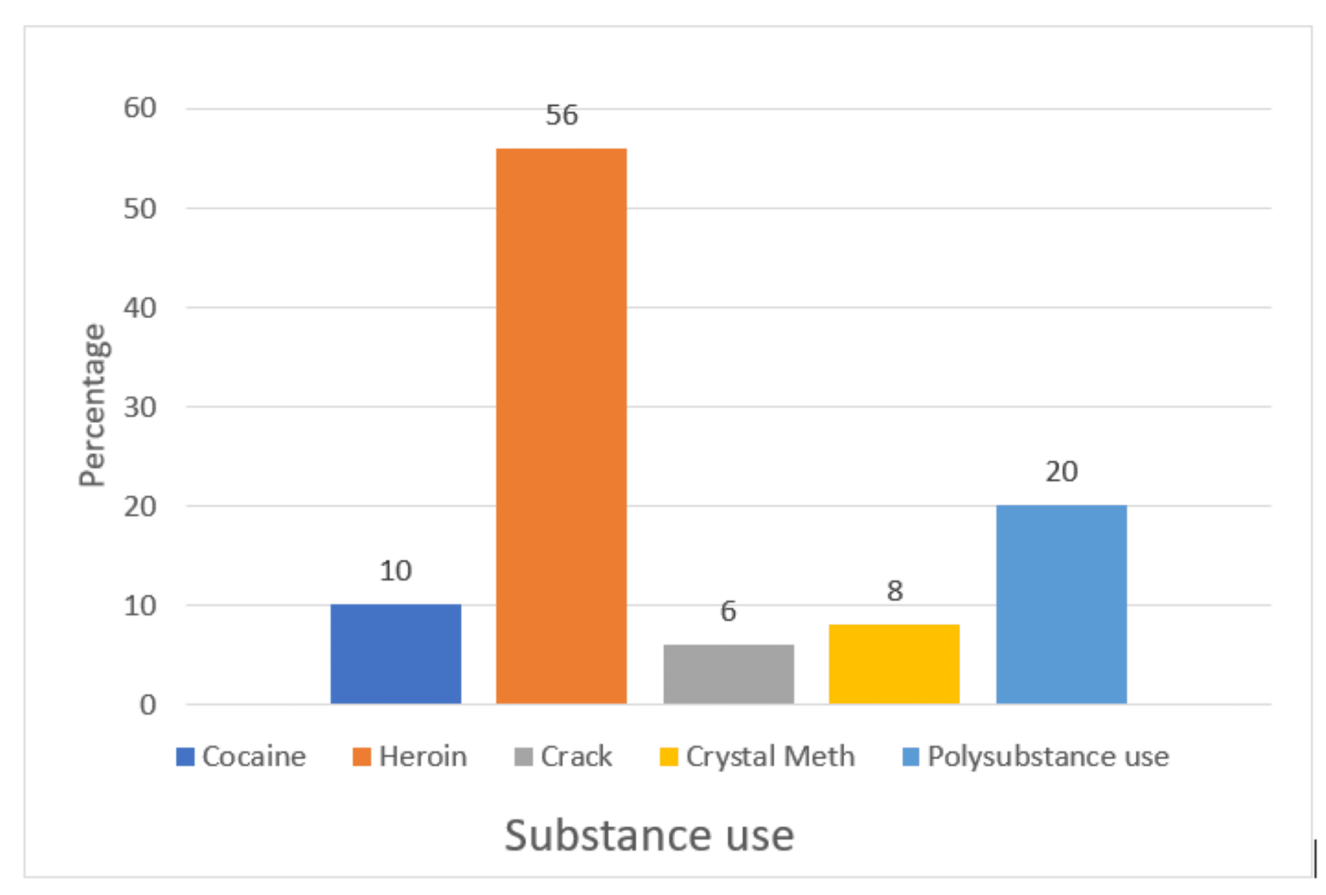
Substances used among study participants

Heroin was the most commonly used drug in the study, with 56% of participants (28 of 50) reporting its use (Figure 1). Polysubstance use was also common, accounting for 20% of the sample. Cocaine, crack, and crystal meth were used by smaller proportions of participants, at 10%, 6%, and 8%, respectively. Despite the high prevalence of substance use among the study population, only 32% of participants (16 of 50) were engaged in OST.

Psychiatric comorbidities and seizure disorders among study participants

**Figure 2:**
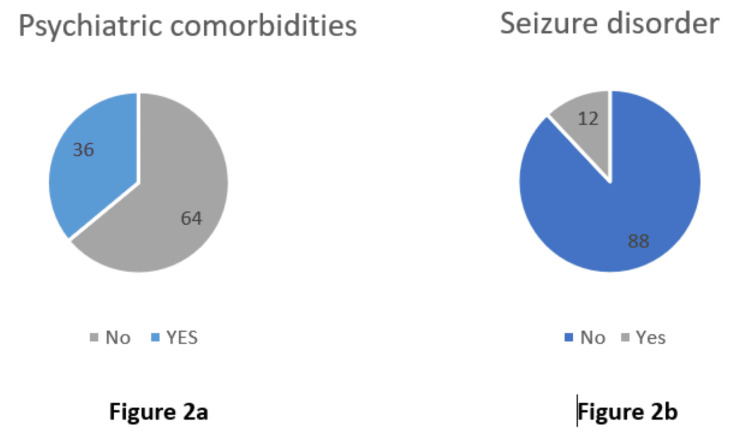
(a) Psychiatric comorbidities among study participants (percentage); (b) seizure disorders among study participants (percentage)

We observed that 36% of participants (18 of 50) had a known psychiatric illness, while 64% (32 of 50) did not (Figure 2a). Additionally, 12% of participants (six of 50) had a known seizure disorder, whereas the remaining 88% (44 of 50) did not (Figure 2b).

Tuberculosis and viral infections among study participants

**Figure 3:**
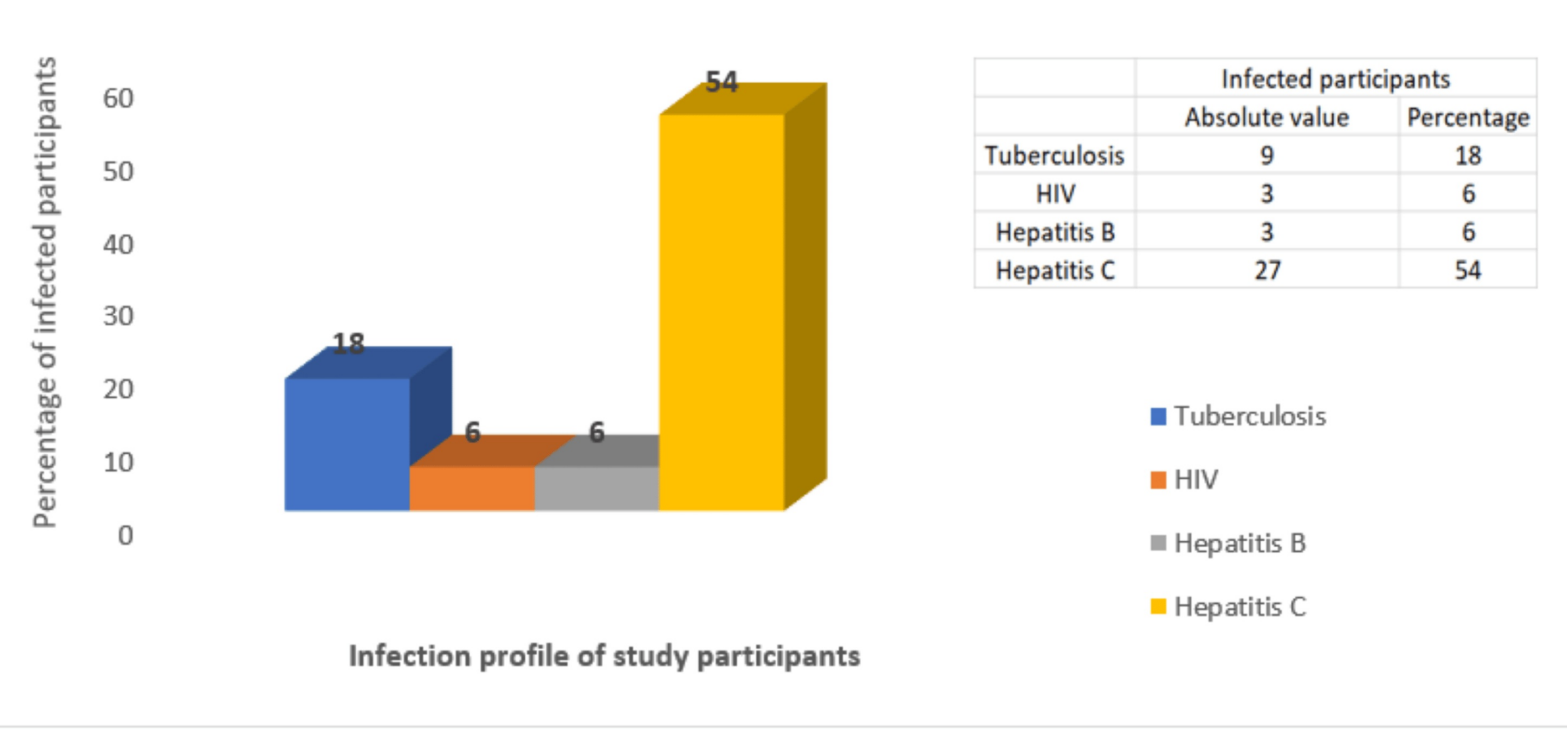
Tuberculosis and viral infection profile among study participants Absolute numbers and percentages of infected participants are provided in the inset table.

The status of tuberculosis and viral infections was assessed among the study participants. Tuberculosis was identified in 18% of participants (nine of 50), of whom 55.5% (five of nine) had active tuberculosis. HIV infection was detected in 6% of participants (three of 50). All HIV-positive participants also had hepatitis B infection. Hepatitis C infection was present in 54% of participants (27 of 50), indicating a high prevalence and a significant health concern in this population. Two participants were identified as triple-positive for HIV, HCV, and HBV infections. Infection profiles are presented in Figure 3.

Diagnoses of infectious disease among study subjects at presentation

The study data indicated that 36% of participants (18 of 50) presented with SSTIs. Osteomyelitis or septic arthritis was observed in 8% of participants (four of 50), pneumonia in 10% (five of 50), and meningitis or brain abscess in 8% (four of 50). Urinary tract infections were present in 18% of participants (nine of 50). IE was definitively diagnosed in 18% of participants (nine of 50) among PWID. The infection profile is summarized in Table 2.

**Table 2:** Diagnoses of infectious diseases among study subjects at presentation IE, infective endocarditis; SSTI, skin and soft tissue infection

Infectious disease	Frequency		Percentage
	Yes	No	
SSTIs	18	32	36
Bone and joint infections	4	46	8
Pneumonia	5	45	10
Meningitis/brain abscess	4	46	8
Urinary tract infections	9	41	18
IE	9	4 1	18

IE among study participants

**Figure 4:**
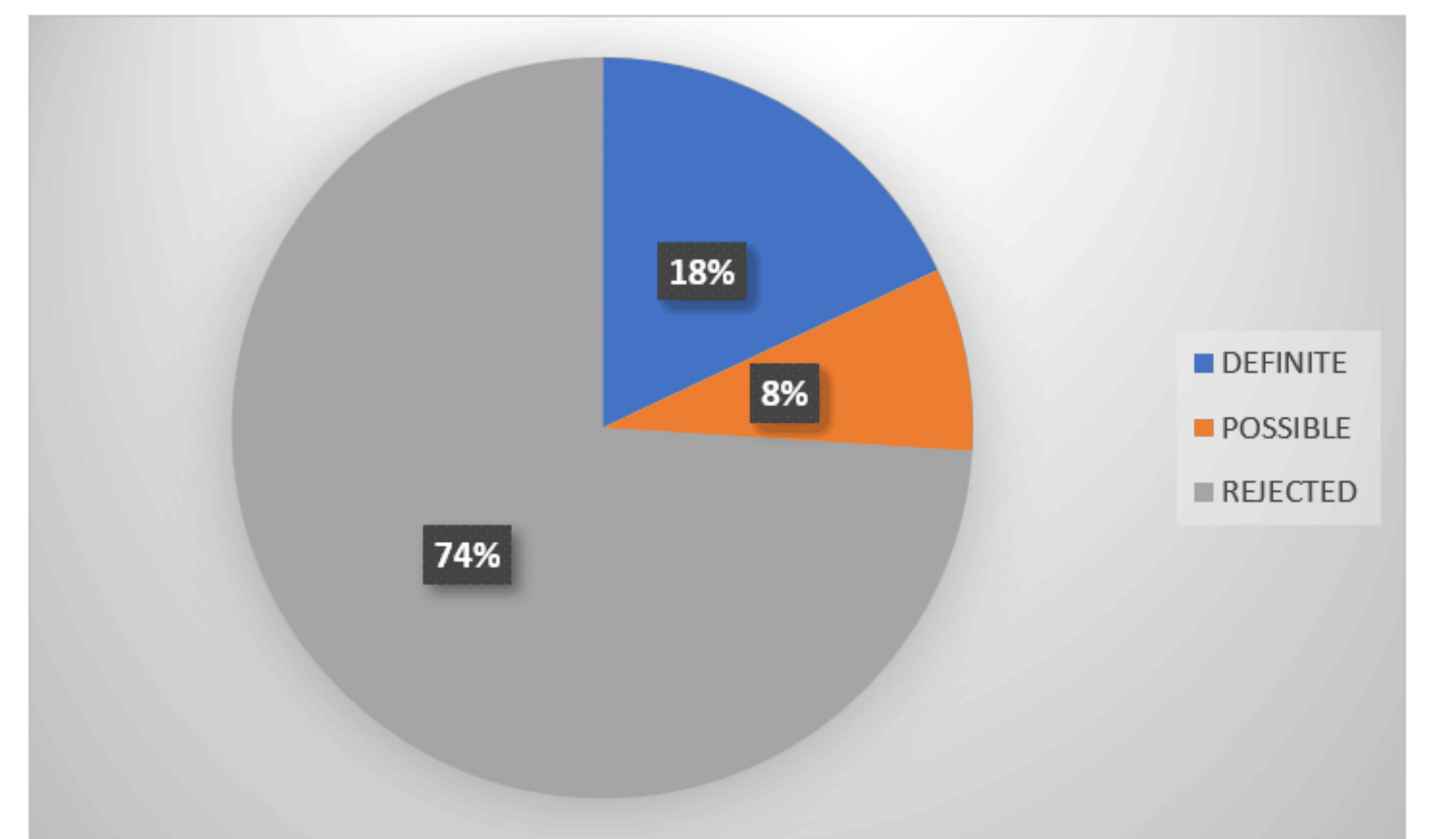
IE among study participants IE, infective endocarditis

Study data on IE among PWID showed that 18% of participants (nine of 50) were definitively diagnosed with IE, while 8% (4 of 50) had a possible diagnosis. In 74% of participants (37 of 50), the diagnosis was rejected based on the European Society of Cardiology 2015 diagnostic criteria for IE, according to major and minor criteria (Figure 4).

Echocardiography findings in relation to IE

Echocardiographic findings suggestive of IE among PWID showed a strong correlation with definitive diagnoses. All nine participants with a definite diagnosis had echocardiographic findings suggestive of IE, while three participants with a possible diagnosis and none of those with a rejected diagnosis had suggestive findings. Among participants with suggestive echocardiography results, tricuspid valve involvement was observed in nine cases, mitral valve involvement in one case, and multivalvular involvement in two cases (Table 3). Overall, nine participants had right-sided valve involvement, and three had left-sided valve involvement.

**Table 3:** Analysis of the site of valve involvement in relation to echocardiography suggestive of IE IE, infective endocarditis

Site of valve involved	Echocardiography suggestive		
	No	Yes	Total
None	38	0	38
Mitral valve	0	1	1
Tricuspid valve	0	9	9
Multivalvular involvement	0	2	2
Total	38	12	50

Organism isolated in relation to IE

Among the nine participants with a definitive diagnosis of IE, various organisms were isolated. Methicillin-resistant *S. aureus *(MRSA) was identified in five cases, while one case each was positive for methicillin-sensitive *S. aureus*, coagulase-negative *Staphylococcus*, *Pseudomonas*, and *Klebsiella*. None of the participants with possible or rejected diagnoses had these organisms isolated (Table 4).

**Table 4:** Organisms isolated in relation to IE CONS, coagulase-negative *Staphylococcus*; IE, infective endocarditis; MRSA, methicillin-resistant *Staphylococcus aureus*; MSSA, methicillin-sensitive *Staphylococcus aureus*

Organism isolated	IE			Total
	Definite	Possible	Rejected	
Culture negative	0	4	37	41
MRSA	5	0	0	5
MSSA	1	0	0	1
CONS	1	0	0	1
*Pseudomonas*	1	0	0	1
*Klebsiella*	1	0	0	1
Total	9	4	37	50

Clinical features at presentation in relation to IE

Study data showed the presence of fever (temperature >38°C) as a minor criterion in relation to the diagnosis of IE. Fever was present in eight of the nine participants with a definite diagnosis, all four participants with a possible diagnosis, and all participants with a rejected diagnosis.

Vascular phenomena (systemic embolism, Janeway lesions, conjunctival hemorrhage, and pulmonary infarction) were observed in seven of the nine participants with a definite diagnosis and in one participant with a possible diagnosis, while none of the participants with a rejected diagnosis exhibited vascular phenomena (Table 5).

**Table 5:** Clinical features at presentation in relation to IE IE, infective endocarditis

Clinical feature		IE		
		Definite	Possible	Rejected
New onset murmur/change in murmur	No	0	2	37
	Yes	9	2	0
Fever temperature >38	No	1	0	22
	Yes	8	4	15
Immunological pneumonia	No	7	4	37
	Yes	2	0	0
Vascular phenomena	No	2	3	37
	Yes	7	1	0

Immunological phenomena (Osler nodes, Roth spots, and glomerulonephritis) were observed in two of the nine participants with a definite diagnosis, while none of the participants with possible or rejected diagnoses exhibited these findings (Table 5).

All nine participants with a definite diagnosis and two participants with a possible diagnosis had a new-onset murmur, whereas none of the participants with a rejected diagnosis had this finding (Table 5).

Complications among study subjects

The most common complications observed included intracardiac complications (abscess, valve perforation, or heart failure) in 12% of participants, systemic embolization in 8%, neurological complications (stroke or brain abscess) in 6%, and pulmonary embolism in 4%.

Among participants with a definitive diagnosis, three of nine had intracardiac complications. Neurological complications were observed in two of nine participants with a definitive diagnosis. Pulmonary embolism occurred in two of nine participants with a definitive diagnosis and in one of four participants with a possible diagnosis. Renal impairment was observed in five of nine participants with a definitive diagnosis. Systemic embolization was reported in four of nine participants with a definitive diagnosis and in one of four participants with a possible diagnosis (Table 6).

**Table 6:** Complications among study subjects IE, infective endocarditis

Complications		IE		
		Definite	Possible	Rejected
Systemic embolism	No	5	4	37
	Yes	4	0	0
Renal impairment	No	4	3	37
	Yes	5	1	0
Pulmonary embolism	No	7	3	37
	Yes	2	1	0
Neurological	No	7	4	37
	Yes	2	0	0
Intracardiac complication	No	6	4	37
	Yes	3	0	0

Clinical outcome among study subjects

Clinical outcomes in relation to IE showed that four participants with a definitive diagnosis were discharged, compared with three of four participants with a possible diagnosis and 29 of 37 participants with a rejected diagnosis (Table 7).

**Table 7:** Clinical outcomes, including in-hospital mortality, among study participants IE, infective endocarditis

Clinical outcome	IE		
	Definite	Possible	Rejected
Discharged	4	3	29
Death	3	0	0
Leave against medical advice	2	1	5
Surgical intervention	1	1	3

Six participants experienced in-hospital mortality due to various causes, including three participants with a definitive diagnosis of IE (Table 7). Among these three deceased participants, complications included brain abscess, valve abscess, and acute mitral regurgitation. Additionally, eight participants left the hospital against medical advice.

Substances used in relation to IE

The study found no significant correlation between the type of substance used and the diagnosis of IE among PWID. Most participants with a definitive diagnosis reported heroin use or polysubstance use, while those with possible diagnoses demonstrated varied substance use patterns. The Pearson chi-square test yielded a value of 7.155 with a p-value of 0.520, indicating no statistical significance (Table 8).

**Table 8:** Statistical analysis of substances used in relation to IE IE, infective endocarditis

Substances used	IE			Total
	Definite	Possible	Rejected	
Cocaine	0	0	5	5
Heroin	7	2	19	28
Crack	0	1	2	3
Crystal meth	0	0	4	4
Polysubstance use	2	1	7	10
Total	9	4	37	50

HIV, hepatitis B, and hepatitis C in relation to IE

Analysis of HIV, hepatitis B, and hepatitis C status in relation to the diagnosis of IE among PWID suggested potential associations. HIV reactivity was observed in two of nine participants with a definitive diagnosis and in one of 37 participants with a rejected diagnosis, while all remaining participants were HIV nonreactive (Table 9). The Pearson chi-square test yielded a value of 5.168 with a p-value of 0.075, indicating that the association was not statistically significant.

**Table 9:** Statistical analysis of HIV, hepatitis B, and hepatitis C in relation to IE IE, infective endocarditis

Infection status		IE			Total
		Definite (9)	Possible (4)	Rejected (37)	
HIV	Nonreactive	7	4	36	47
	Reactive	2	0	1	3
Hepatitis B	Nonreactive	6	4	37	47
	Reactive	3	0	0	3
Hepatitis C	Nonreactive	1	2	20	23
	Reactive	8	2	17	27

Hepatitis B reactivity was observed in three of nine participants with a definitive diagnosis, while all other participants were nonreactive. The Pearson chi-square test yielded a value of 14.539 with a p-value of 0.001, indicating strong statistical significance.

Hepatitis C reactivity was observed in eight of nine participants with a definitive diagnosis and in two of four participants with a possible diagnosis, compared with 17 of 37 participants with a rejected diagnosis. The Pearson chi-square test yielded a value of 5.402 with a p-value of 0.067, indicating borderline statistical significance.

## Discussion

A mean age of 25.94 years was observed in our study, consistent with global trends showing increasing representation of younger age groups among PWID populations. A marked male predominance was also observed, which aligns with findings from both Indian and international studies.

Heroin emerged as the predominant substance used, with 56% of participants reporting its use. This finding is consistent with global trends; for example, the United Nations Office on Drugs and Crime (UNODC, 2020) reports that heroin is the most commonly injected opioid worldwide. Similarly, previous studies have shown that heroin remains the primary drug of choice among PWID in many countries [20], including India, where the National Drug Dependence Treatment Centre (NDDTC) has reported a high prevalence of heroin use among PWID in urban areas [21].

PWID demonstrated a high prevalence of hepatitis C infection in our study, with a positivity rate of 54%, consistent with international reports [22]. Chronic HCV infection is associated with endothelial dysfunction and immune dysregulation, which may increase the risk of IE in this population. The coexistence of high HCV prevalence and SSTIs among PWID necessitates a multidisciplinary approach to health care. These conditions reflect high-risk behaviors and may predispose individuals to serious infections such as IE. Although traditional screening methods for IE remain unchanged, the frequent presence of HCV among PWID may worsen IE-related morbidity and outcomes due to co-infection. Given that SSTIs are common among PWID and may precede or exacerbate IE, vigilant screening for IE is recommended in PWID presenting with SSTIs.

SSTIs were prevalent in this study, with 36% of participants reporting such infections. Contributing factors include poor hygiene practices, the use of contaminated injection equipment, and compromised immune function. Similar or higher prevalence rates have been reported in studies from other parts of India. For example, Lavender and McCarron reported that approximately 28% of PWID experience SSTIs, ranging from localized cellulitis and abscesses to severe necrotizing fasciitis [22].

In our study, 18% of participants (nine of 50) were definitively diagnosed with IE based on the Modified Duke criteria (2000). These findings are consistent with other studies examining the prevalence of IE among PWID. Goyal et al. reported that 40.6% of IE cases in their study were associated with IV drug use, whereas rheumatic heart disease accounted for only 9% of cases [23]. This underscores the substantial contribution of IDU to the burden of IE compared with other predisposing conditions. However, reported prevalence rates of IE associated with IV drug use vary across regions, with estimates of 25.34% in North America and 21.46% in Europe [24].

Fever was the most common presenting symptom, observed in 88.8% of participants with a definitive diagnosis of IE. Vascular phenomena, including embolism, Janeway lesions, and conjunctival hemorrhage, showed a strong correlation with IE diagnoses. Immunological manifestations, such as glomerulonephritis and Osler nodes, also contributed to the diagnosis of IE.

Echocardiographic findings suggestive of IE demonstrated a strong correlation with definitive diagnoses in this cohort. All nine participants with a definitive diagnosis showed echocardiographic evidence consistent with IE, including valvular vegetations, abscesses, and other characteristic endocardial abnormalities. Among participants with suggestive echocardiographic findings, 75% had tricuspid valve involvement, 8.3% had mitral valve involvement, and 16.6% had multivalvular involvement. Comparable findings have been reported in other studies, with tricuspid valve involvement observed in 68.5% of IE cases among IVDU, followed by mitral valve involvement in 22.2% of cases [23].

The microbiological profile revealed a diverse range of causative pathogens, reflecting the complex nature of IE in this population. Gram-positive organisms predominated (77.7%), with *S. aureus *accounting for the majority of cases. This finding reinforces the established association between *S. aureus*, SSTIs, and IDU. Similarly, previous studies have reported MRSA accounting for 42.6% of IE cases (p < 0.003) and *Pseudomonas*-related IE accounting for 18.5% (p < 0.003) among IVDU.

Common complications observed in this study included intracardiac complications such as abscess formation, valve perforation, and heart failure (12%); systemic embolization (8%); neurological complications, including stroke and brain abscess (6%); and pulmonary embolism (4%). These findings highlight the high morbidity associated with IE among PWID and underscore the need for prompt and effective management. Similar studies have reported systemic embolization (14%) as the most common complication of IE among PWID, followed by ischemic stroke (4%) and intracardiac complications (2%). Lavender and McCarron reported that pulmonary septic embolism and pulmonary infarction are commonly associated with right-sided endocarditis and should be considered in cases of tricuspid valve involvement [22]. Another study documented pulmonary infarction in 26.23% of cases, pulmonary edema in 14.75%, and intracranial complications in 6.56% of PWID with IE [25].

As a single-center, hospital-based study, the findings may not be representative of all PWID in the community, particularly those who do not seek medical care or who present with milder infections. Additionally, the relatively small sample size of 50 participants limits the generalizability of the results. Validation of these findings in larger cohorts would improve the precision and robustness of the study conclusions.

## Conclusions

This study enhances our understanding of infections among PWID. Addressing gaps in health-seeking behavior is critical to improving treatment adherence and mitigating adverse outcomes in this population. The high prevalence of IE amid SSTIs and HCV infection highlights the urgent need for enhanced screening using echocardiography and blood cultures in young, heroin-using PWID presenting with fever or vascular phenomena. Targeted interventions, including increased uptake of OST and HCV treatment, may help prevent progression from SSTIs to IE, thereby reducing mortality and complications. Future research should focus on larger cohorts and longitudinal studies to validate and expand upon these findings.
